# Supplementary material for: Are carbon emissions trading and green financial instruments synergistic? -Comprehensive quantitative research based on content analysis
Source: PLoS One. 2024 Mar 7;19(3):e0298601. doi: 10.1371/journal.pone.0298601 (PMC10919601; doi:10.1371/journal.pone.0298601)
Supplement: S1 Data — (ZIP) [file pone.0298601.s001.zip › Data/地级市Z_F工作报告.zip!/║╙▒▒/╠╞╔╜╩╨/└┤╫╘╣1⁄2╓┌║┼í╛┬φ┐╦╩2╛▌═°í┐_2007.docx]

一、2006年政府工作回顾

刚刚过去的一年是我市发展历史上不平凡的一年。这一年，胡锦涛总书记亲临唐山视察，对我们提出了建设繁荣文明和谐新唐山的殷切希望，全市人民受到了极大鼓舞。这一年，我们成功举办了抗震三十周年纪念活动，新唐山以非凡的建设成就、巨大的发展潜力和英雄城市的独特魅力，引起了海内外各界极大关注。这一年，全市人民在市委的领导下，认真贯彻落实科学发展观，团结奋斗，开拓进取，经济社会发展取得了令人鼓舞的新成绩，实现了“十一五”良好开局。

——扎实推进结构调整，全市经济继续保持了平稳较快发展的良好势头。预计全市地区生产总值达到2362亿元，比上年增长14.6%;全部财政收入264.3亿元，增长16.7%，其中地方一般预算收入93.4亿元，增长20.3%。积极、有作为地贯彻落实国家宏观调控政策，加快改造传统产业，提升产业集中度，工业经济运行质量进一步提高。全市规模以上工业增加值完成960亿元、利润260亿元，分别增长23%和36%，其中钢铁、建材、能源、装备制造和化工五大产业增加值、利润分别占规模以上工业的81.3%和79%。着力推进钢铁工业调整整合，全市钢铁企业户数由年初的30家整合至20家，淘汰了一批落后产能，钢铁工业整体素质显著提升。服务业发展步伐加快，增加值增长14.6%。全社会消费品零售总额增长15%，居民消费价格指数为101.3%。投资稳定增长，重点建设步伐加快。全社会固定资产投资完成765亿元，增长20.3%。其中市以上重点项目完成投资281亿元，增长17%。创新能力进一步增强，科技进步对经济增长贡献率达到56.5%。狠抓节能降耗，积极发展循环经济，切实转变经济增长方式。预计全年重点用能单位万元产值综合能耗同比下降4.4%。

——大力实施生产力布局向沿海推进战略，曹妃甸工业区建设取得重大突破。抓住曹妃甸被国家列入“十一五”规划和首批循环经济试点园区的历史机遇，坚持以规划为先导，以项目为载体，突出港口建设，强化基础设施，狠抓产业集聚，举全市之力推进曹妃甸工业区开发建设，全年完成投资100亿元。唐山港规划、曹妃甸循环经济示范区产业发展规划、曹妃甸工业区“十一五”发展规划等规划编制工作取得重要成果。唐山港货运量突破5000万吨，其中曹妃甸25万吨级矿石码头达到1100万吨。曹妃甸原油码头、煤炭码头一期获国家核准并投入建设; LNG码头、挖入式港池、工作船码头、通用码头建设按计划推进。迁曹铁路建成通车，供水工程建成通水，唐曹高速开工建设，吹填造地完成40平方公里，配套设施日趋完备，为大规模开发建设准备了必要条件。精品钢材基地、华润电厂具备开工条件，大型钢构、修造船、海水淡化等一批项目前期工作取得重要进展。海港开发区、南堡开发区建设步伐加快，预计地区生产总值分别较上年增长53.7%和53%，财政收入分别增长35%和26.9%。

——狠抓农村经济和各项社会事业发展，新农村建设扎实起步。全面落实各项惠农政策，农业税全部免除。粮食总产达到288万吨，是近五年来的最高产量。乳业、瘦肉型猪、果菜、板栗、花生、水产品六大龙型经济健康发展，全市农业产业化经营率达到57%。农业结构继续得到调整和优化，畜牧水产业比重达到50%，被评为“全国牛奶生产强市”。围绕加强农村教育、卫生、社保、文明生态村建设，进一步加大投入力度。改造农村中小学陈旧校舍13.4万平方米,享受免除杂费的贫困生比例由上年的35%提高到78.4%，迁安、遵化、迁西、丰南全部免除义务教育阶段学生学杂费。乡镇卫生院(室)基础设施和标准化建设基本到位，新型农村合作医疗参保农民达到447.9万人，覆盖率达到91.8%。整合改造农村敬老院35所，农村最低生活保障标准由每人每年800元提高到1000元。新创建文明生态村600个，全市已有2528个行政村达到文明生态村创建要求，占总数的44%。

——深入推进改革开放，经济社会发展活力进一步增强。国有企业改革进一步深化，列入两年改制重点的33户困难企业，已有13户完成改制。国有资产监管体系不断完善，行政管理体制改革继续深化，公务员登记工作基本完成，事业单位改革稳步推进。全市实际利用外资5亿美元，增长8.5%;进出口总额达到33亿美元，其中出口18.5亿美元，分别增长24.5%和52.5%。不断加强与京津等地的合作，达成一批重要合作项目，引进市外资金135亿元。成功举办了第九届唐山中国陶瓷博览会。民营经济健康发展，占全市地区生产总值的比重由上年的60.5%上升到63.5%。

——深入实施城市化战略，城乡建设步伐加快。城市基础设施完成投资146.2亿元，是近年来投入最多的一年。唐承高速一期完工，沿海高速等一批重大项目扎实推进。新建、改造城市道路52条，佳华煤气输唐管线等一批重点市政公用设施投入使用。扩供煤气8000户，新增集中供热面积96万平方米，新增公交车辆328部。稳步推进住宅建设，全市商品房竣工225万平方米，经济适用住房竣工14.7万平方米。深入实施绿化、美化、亮化、净化和畅通工程，城市面貌明显改观。县(市)城和小城镇建设步伐加快，城市功能和人居环境进一步提升。大力实施“村村通”工程，在全市实现公路“村村通”的基础上，提前一年实现农村客运“村村通”，成为全省率先实现“双通”目标的城市。加强环境保护，改善环境质量。2006年城市空气质量二级及优于二级的天数达到301天。预计全市城镇化率达到48%，比上年提高1.9个百分点。

——加快发展各项社会事业，促进了社会和谐进步。以纪念抗震三十周年为契机，深入开展抗震精神再教育活动和“八荣八耻”社会主义荣辱观道德实践活动，全市人民的凝聚力进一步增强，社会文明程度不断提高。教育事业健康发展。着力提高基础教育水平，初中巩固率达到98%，初中毕业生升学率达到86.2%，在全省率先实现基本普及高中阶段教育;加快发展高等教育，理工大学新校区、煤炭医学院新校区、师范学院北校区和唐山学院北校区建设全面启动。卫生事业水平不断提高。公共卫生和医疗服务体系不断完善，市县两级疾控中心全部投入使用，城市社区卫生服务得到加强。文化事业进一步繁荣，“俏夕阳”、“红星谣”等一批优秀作品和剧目获国家大奖，成功举办了第五届中国评剧艺术节和第五届唐山社区文化艺术节。全民健身活动广泛开展，省十二届运动会筹备工作进展顺利。在省运会已结束的19个项目比赛中，我市代表团夺得金牌108枚，创1983年以来竞技体育最好成绩。人口增长得到有效控制，人口自然增长率保持在5.45‰。社区建设、社会福利、社会救济、优抚安置等民政事业取得新的进步。土地、矿产、海洋等自然资源保护和合理开发利用取得新进展。广播电视、民族宗教、外事侨务、防震减灾、气象、人防、档案等各项工作也都取得了新的成绩。

——下大力解决就业、社会保障等事关群众切身利益的问题，城乡居民生活质量不断提高。更加关注民本民生，让改革发展的成果惠及全市人民。全市城镇居民人均可支配收入达到12376元，农民人均纯收入达到5155元，分别增长18%和12.5%。消费结构继续升级，住房、汽车、通信、文娱、旅游消费持续增长。高度重视就业和再就业工作，全年城镇新增就业6.2万人，3.2万名下岗失业人员实现了再就业。城镇登记失业率为4.35%。完善社会保障体系，养老、失业、工伤和医疗等保险扩面工作扎实推进，离退休人员基本养老金和失业人员失业保险金按时足额发放。城乡居民最低生活保障实现应保尽保，为3795户低收入家庭解决了住房困难。市政府为群众办的25件实事全部如期完成。

——着力提高行政服务水平，发展环境进一步优化。坚持执政为民，全面履行职能，进一步健全完善了科学民主决策机制、依法行政机制和行政监督机制，大力加强制度建设和机关效能建设，促进了政府服务和管理水平的提高。坚持并完善了向人大报告工作、向政协通报情况制度，自觉接受人大法律监督和工作监督，重视发挥政协的政治协商、民主监督和参政议政作用。承办人大代表意见建议119件，政协委员提案444件，全部按时办结，满意率达到97%。积极推进依法治市，普法教育、基层民主政治建设不断深入。扎实推进“阳光行动”、行风评议，反腐败斗争取得阶段性成果。强化社会治安综合治理，加强安全生产工作，维护了全市政治安定和社会稳定。下大力创优发展环境，去年先后被评为“中国投资环境50优城市”、“中国最具投资潜力十强城市”, 并首次进入福布斯中国大陆最佳商业城市榜。

各位代表，经过全市上下的共同努力，我们胜利完成了十二届人大四次会议确定的各项目标任务，不仅巩固和扩大了我市经济社会持续快速发展的良好势头，而且使今后实现跨越式发展的基础更加牢固。这是在市委的领导下，全市人民同心同德、锐意进取、艰苦奋斗的结果，是市人大、市政协监督帮助和全市各界人士关心支持的结果。在此，我代表市政府，向全市工人、农民、知识分子、机关干部、公安政法干警、驻唐部队、武警官兵以及社会各界人士，表示崇高的敬意和衷心的感谢!在充分肯定成绩的同时，我们也清醒地认识到，政府工作中还存在着许多问题和不足，前进道路上还面临着一些突出矛盾和困难。主要是：作为传统资源型城市，我市经济结构性矛盾依然比较突出，特别是钢铁产业调整整合任务艰巨;经济增长方式粗放的状况还没有根本改变，节能降耗和环境保护工作任重道远;服务业发展滞后，消费拉动作用还没有充分发挥出来;开放型经济竞争力不强，科技创新能力亟待提高;城市建设和管理与经济发展水平和人民群众生产生活需求不相适应，城市形象、交通、人居等诸多方面不尽如人意;城乡居民收入水平和社会保障水平还不高，就业压力加大，部分低收入家庭生活还比较困难。政府职能转变步伐不快，公共服务和社会管理工作有待改进和加强。一些政府机关干部思想观念还不适应新形势新任务的要求，责任感、事业心不强，办事效率不高，服务不到位。对这些问题，我们一定引起高度重视，在今后工作中认真加以解决。各位代表，经过全市人民这些年的不懈努力，我市无论是综合经济实力，还是内外发展环境都发生了巨大变化。在我市改革发展关键时刻，胡锦涛总书记亲临唐山视察，殷切期望我们建设繁荣文明和谐新唐山，把曹妃甸建成科学发展示范区;省第七次党代会鲜明地提出了建设沿海经济社会发展强省的宏伟战略，并要求唐山在打造沿海经济隆起带中发挥带头作用、在冀东城市群建设中发挥龙头作用、在建设社会主义新农村中发挥示范作用，在建设沿海经济社会发展强省中当好“领头羊”。综观全局，总体形势于我市加快发展十分有利，特别是环渤海经济的迅速崛起和京津冀都市圈规划的制定实施，使我市的产业、区位、资源和港口优势更加凸显。我们完全有条件发展得再好一些、再快一些。我们必须倍加珍惜难得的历史机遇，倍加珍惜全市上下政通人和、团结奋进的大好局面，把中央和省的重大决策部署与唐山发展高度统一起来，坚定信心，把握大势，找准定位，抢抓新机遇，建设新唐山，实现新跨越，创造新奇迹!

二、2007年经济社会发展总体思路和奋斗目标

2007年是实施“十一五”规划的关键一年，也是落实胡锦涛总书记对唐山重要指示和省委要求我市当好“领头羊”的第一年。刚刚结束的市委八届三次全会，深刻分析了我市经济社会发展形势，作出了我市正处于经济社会加速转型、全面小康和现代化建设加速推进的新阶段的科学判断，明确提出了未来五到十年把唐山建设成为文化名城、经济强城、宜居靓城、滨海新城的新目标，并对当前和今后一个时期的工作进行了战略部署。根据市委八届三次全会精神，今年我市国民经济和社会发展总体要求是：坚持以邓小平理论、“三个代表”重要思想和科学发展观为指导，以建设科学发展示范区为主导，以“抢抓新机遇、建设新唐山”为主题，以依托大港、走向海洋、跨越发展为基本方略，紧紧围绕实现更好更快发展、建设和谐唐山两大主要任务，更加着力推进发展模式转变和资源型城市转型;更加着力加快经济结构的战略性调整和产业优化升级;更加着力保护生态环境和节约资源能源;更加着力深化改革扩大开放和体制、机制、政策创新;更加着力推动城市与农村、沿海与腹地的良性互动和区域协调发展;更加着力改善民生、提高人民群众的幸福指数，扎实推进经济、政治、文化、社会建设，在科学发展的道路上实现新跨越，加快建设繁荣文明和谐的新唐山。全市经济社会发展的主要预期目标是：地区生产总值增长13%以上，单位生产总值能耗降低4.4%;全部财政收入增长13.5%;全社会固定资产投资增长23%以上;城镇居民人均可支配收入增长10%，农民人均纯收入增长8%;新增就业岗位6万个以上，城镇登记失业率控制在4.5%左右。

做好今年的工作，最根本的是以科学发展观统领经济社会发展全局，最关键的是从实际出发，重点突破，带动全局。必须牢牢把握以下原则：一是坚持把“抢抓新机遇、建设新唐山”作为主题。进一步解放思想，与时俱进，强化沿海意识，以更宽的眼界、更高的站位，科学谋划唐山未来发展;立足当前，着眼长远，善于把握发展机遇，勇于创新发展思路，大胆探索发展途径;防骄破满，自我加压，负重奋进，真正当好建设沿海经济社会发展强省“领头羊”。二是坚持把推进资源型城市转型作为战略任务。以建设科学发展示范区为主导，加快由资源主导型经济向沿海开放型经济转变，依靠新区带动老区，依靠曹妃甸搞大进大出，走新型工业化道路;加快由相对封闭的城乡二元结构向开放的城乡一体化转变，实现城乡经济社会的协调发展;加快由内陆型城市向滨海宜居城市转变，打造真正意义上的现代化滨海城市。三是坚持把推进结构调整、转变增长方式，实现更好更快发展作为着力点。围绕推进曹妃甸新区开发建设，打造沿海经济隆起带，加快调整优化生产力布局;狠抓经济结构战略性调整，着力打造精品钢材、先进制造业、精细化工、现代农业、现代物流、文化旅游、高新技术等七大产业链，不断提高经济的整体实力和核心竞争力。正确处理“好”与“快”的关系，力求在争好的前提下保持较快的发展速度，努力做到好中求快，快中见好。四是坚持把改革开放和创新作为根本动力。要以更大的决心破除各种思想和体制障碍，加快体制机制创新;以更大的魄力推进双向开放，拓展发展空间;以更大的力度推进科技创新，增强科技进步对经济社会发展的引领和支撑作用。五是坚持把提高人民群众的幸福指数作为出发点和落脚点。把人民群众利益放在首位，以解决人民群众最关心、最直接、最现实的利益问题为重点，深入实施扩大就业、社保扩面、城镇安居、教育扶助、全民健康、文化繁荣、蓝天碧水、政务创优、诚信平安、民主参与“十大幸福工程”，尽心竭力为群众谋利益、办实事，让人民群众在改革发展中得到更多实惠。

三、促进经济更好更快发展

始终坚持以科学发展观为统领，全面贯彻“在好中求快、在快中见好”的总体要求，突出重点，抓住关键，攻坚克难，努力走出一条集约发展、循环发展、创新发展、开放发展、和谐发展、协同发展之路。

(一)全力打造曹妃甸新区。认真贯彻落实胡锦涛总书记和省委、省政府对曹妃甸开发建设的重要指示和工作要求，高起点、高质量、高水平把曹妃甸新区规划好、建设好、使用好，努力建设成为东北亚航运枢纽中心、国际性精品钢材生产交易中心、中国重型装备制造业生产基地、世界级化学工业生产基地。从长远发展战略出发，将全市沿海九个行政单位纳入曹妃甸新区的规划范畴，抓紧启动曹妃甸新区总体规划和各专项规划的编制，统筹产业分工与资源配置和城镇布局。科学设定产业准入门槛，积极引进战略投资者参与曹妃甸新区建设，推动重大项目产业聚集。坚持临海与腹地互动，借沿海优势，推进区域经济融合。推进体制机制创新，探索建立利益共享机制，逐步建立统一、协调、高效的曹妃甸新区管理体制。曹妃甸工业区是曹妃甸新区的核心，今年确保完成投资300亿元以上。一是按照“科学发展示范区”要求，尽快完成唐山港总体规划，高起点编制曹妃甸港口、港区、港城规划及专项规划。二是推进基础设施建设。加快煤炭码头、原油码头、LNG码头和一号挖入式港池建设，确保原油码头年底通航，煤炭码头2008年建成，LNG码头年内开工，一号挖入式港池部分码头投入使用;抓好25万吨级矿石码头运营，全年吞吐量达到1600万吨以上。加快造地工程进度，配套抓好唐曹高速、司曹铁路、迁曹铁路运煤专线、净水厂、污水处理厂等重大基础设施建设。三是加快产业突破。确保首钢精品钢材基地、华润2×30万千瓦热电机组、二十二冶钢构等一批项目开工建设，积极争取修造船、直升机、400万吨水泥超细粉等一批项目尽早开工，加快推进华北原油储备基地、大型石油炼化、矿石物流等一批重大项目的前期工作，积极谋划一批符合曹妃甸产业布局发展的新项目，并力争取得实质性进展。

(二)强力推进产业结构特别是钢铁结构调整。按照资源深度利用、产品中高端延伸、产业相互融合、区域优势互补的思路，以优势主导产业为依托，加快培育壮大产业链经济。坚持把打造精品钢材产业链作为工业结构调整的重中之重，加快由钢铁大市向钢铁强市转变。按照有关政策要求，坚决禁止新增产能，坚决淘汰落后产能，坚决落实环保措施;以曹妃甸大钢、唐钢、国丰、津西等大型企业为骨干，加快推进钢铁行业调整整合，确保2007年底钢铁企业户数整合到10家以内;着力抓好钢铁品种结构调整和深加工，力争板带比达到60%。坚持把发展先进装备制造业作为延伸钢铁产业链、增强区域经济竞争力的重大战略和重要突破口，编制发展规划，加强政策引导，加紧谋划建设一批重大装备制造业项目，加快建设北方重型装备制造业基地。立足发挥比较优势，不失时机地推进煤化、盐化产业发展，加快推进石化项目前期工作。大力发展高新技术产业，壮大电子信息、机电一体化、生物技术、新材料等高新技术产业群体。优化企业和产品结构，大力实施名牌战略，引导企业打造知名品牌，增强市场竞争能力。着力培育精品钢材、陶瓷、食品等优势集群品牌。重点扶植一批产品争创全国驰名商标和河北著名商标，积极做好中国名牌、中国畅销品牌和河北出口名牌的申报工作。

(三)大力发展服务业。加快服务业发展，是我市优化结构、促进消费、扩大就业、拉动经济增长、提高现代化水平的重大战略。坚持市场化、社会化和产业化发展方向，抓住生产力布局调整和居民消费全面升级的重要机遇，突出重点领域和重点行业，加大政策扶持和投入力度，力促服务业扩容提速，确保服务业增加值增长14%以上。一是结合生产力布局向沿海推进战略，以发展生产性服务业为突破口，大力培育以海洋运输为龙头的现代物流产业链，积极推进曹妃甸国际物流园区、京唐港区物流中心等一批重点项目，促进沿海二三产业协调发展。二是结合增强消费拉动，鼓励发展金融、保险、文体、房地产、中介咨询、社区服务等需求潜力大、劳动密集的产业，改造提升商贸流通、餐饮娱乐等传统服务业。整合旅游资源，打造旅游品牌，抓好遵化汤泉皇家旅游度假区、乐亭新戴河、迁西景忠山等一批重点项目建设，加快旅游业发展步伐。三是结合新农村建设，以农资供应、农副产品运销、农村社会事业和农民消费为重点，加快拓展农村服务业。四是建立健全服务业政策支持体系，进一步扩大市县两级服务业发展引导资金规模，对关键领域、薄弱环节和新兴业态给予支持;认真清理制约服务业发展的不合理规定，简化行政审批，消除影响服务业发展的体制性障碍。

(四)保持固定资产投资合理增长。认真贯彻落实国家宏观调控政策，围绕产业结构调整，创新发展理念，优化投资结构，增强经济发展后劲和可持续发展能力。全年安排基础设施、先进制造业、高新技术产业、现代服务业和农业产业化等领域重点项目90项，年度投资计划350亿元，比去年增加70亿元。突出抓好冀东油田勘探开发等40个续建项目建设进度，力促唐山蓝欣镀膜玻璃生产线等40个计划开工项目开工建设，确保20个项目年内竣工;抓好大唐王滩电厂二期等10个重大项目的前期工作，争取早日开工;积极谋划一批符合国家产业政策、产业关联度高、带动能力强的重大项目，不断充实项目库。高度重视民间投资和中小企业投资，下大力抓好与大项目相配套、延伸产业链条、有利于社会就业的中小项目。积极采取土地置换、盘活存量、集约用地、争取国家和省支持等多种措施，最大限度地缓解项目用地紧张局面。围绕土地集约经营、产业链条延伸和资源循环利用，大力发展园区经济。抓好高新区日资工业园、玉田电子工业园等一批重点园区建设，促进产业集群发展。深化投融资体制改革，积极发展资本市场，鼓励企业直接融资，健全多元化投入机制。

(五)高度重视并切实抓好节能降耗和环境保护。把节能降耗和环境保护作为转变经济增长方式的突破口，进一步强化措施，加大力度。狠抓节能降耗。落实单位地区生产总值能耗公报制度，实行节能工作实绩评价考核制和问责制，在冶金、建材、化工等高耗能行业实施10大节能工程，推广30项节能技术，推进耗能大市向节能大市转变。按照“减量化、再利用、资源化”的原则，加快发展循环经济，重点抓好曹妃甸循环经济示范区和唐钢、开滦、冀东水泥等大型企业循环经济试点建设。力争城市工业用水重复利用率达到80%以上，高炉煤气回收利用率达到70%以上，粉煤灰、煤矸石利用率分别达到50%和80%以上。充分利用价格杠杆和财税、政府采购等政策，促进能源节约和有效利用。在全社会倡导健康文明的生产生活方式和消费方式，创建节约型社会。切实加强环境保护。深入实施“蓝天碧水”工程，健全完善环境保护决策机制、考核机制、准入机制和责任追究机制，严格落实建设项目环保“三同时”、排放许可和环境影响评价制度，实行“环保一票否决”，建立环境保护长效机制。切实加大环保执法力度，大力整治水污染、大气污染和固体废弃物污染，对市区内各种重污染企业作出规划，分类施治，坚决“关闭一批、搬迁一批、管好一批”，对环境违法行为发现一起、查处一起。在加强日常监管的同时，把每年3月份作为“全市环保联合执法专项行动月”，对环保违法行为进行拉网式检查整治。严格落实污染物排放总量控制，抓好电力行业脱硫、钢铁行业除尘等34 项重点污染治理项目，全市主要污染物排放总量减少3%。积极发展环保产业。实行全市空气质量周报等环境保护信息公开发布制度，扩大公众环境保护知情权，提高公众环保意识，在全社会形成关心支持环境保护的良好氛围。健全环境突发事件应急机制，保障公众生命健康和财产安全。

(六)加快推进新农村建设。深入贯彻工业反哺农业、城市支持农村和多予、少取、放活的方针，突出“一个中心”、“三大保障”，以统筹城乡发展为基本途径，以县域经济为重要支撑，以创建文明生态村为载体，推动新农村建设健康发展。牢牢把握农村经济这个中心，促进农民持续增收。巩固完善各项支农惠农政策。调整农业结构，提升产业层次，大力发展高产、优质、高效、生态、安全农业，加快构筑现代农业产业体系。抓好特色农产品基地建设，积极引进国内外优良种、养、加技术，推行标准化和无公害、绿色农产品生产，培育农业品牌。扩大农产品出口，发展外向型农业。健全农技推广、良种繁育、动植物防疫、农产品质量检测、农业风险防范等服务体系。以打造现代农业产业链为方向，继续壮大乳业、瘦肉型猪、果菜、板栗、花生、水产品六大龙型经济，积极培育肉牛羊、甘薯、花卉、食用菌和林产品加工等五大新兴产业。抓好滦南蒙牛二期等58个投资1000万元以上重点农业产业化项目建设，全市农业产业化经营率达到59%。加强农村二三产业发展，大力发展劳务经济，改善农民进城就业环境，努力增加农民的工资性收入。全年转移农村劳动力15万人次。围绕培育高素质新型农民，切实加强农民教育培训，提高农民的生产技能和劳动就业能力。加快发展农村各项社会事业，建立健全“三大保障”。完善农民基本生活保障：健全农村最低生活保障和社会救助制度，积极推进失地农民养老保险，新改造农村敬老院33所。强化农村基础设施和社会事业保障：新创建文明生态村500个，改造县乡村道路520公里，新改造农村中小学陈旧校舍8万平方米，全部免除农村义务教育阶段学生学杂费，进一步健全完善农村合作医疗制度，实施“万名医师支援农村卫生”工程。健全新农村建设体制保障:以乡镇机构、农村义务教育和县乡财政体制改革为主要内容,加快推进农村综合改革。遵循客观规律，积极探索符合实际的新农村建设途径,尊重农民意愿，发挥农民主体作用，防止形式主义和损害农民利益的问题发生。建立并形成新农村建设稳定的投入增长机制。市县两级财政每年对农业总投入的增长幅度要高于财政经常性收入的增长幅度，切实做到每年财政支农投入的增量高于上年，固定资产投资用于农村的增量高于上年，土地出让收入用于农村建设的增量高于上年。

(七)全面深化改革扩大开放。解决发展中的各种突出矛盾和问题，根本出路在于改革开放。必须进一步加强对改革开放工作的指导协调，努力在一些关键问题上实现突破。在改革上，重点抓好31家市属国有和集体困难企业的产权制度改革，力争年内全面完成。加快大型企业股份制改革步伐，优化股权结构，积极发展混合所有制经济。完善国有资产监管体制，加强国有企业监事会工作，确保国有资产保值增值。深化行政管理体制改革，加快政府职能转变。加强县乡管理体制改革，支持有条件的县(市)探索扩权强镇。健全完善公共财政体系和绩效评价体系，严格收支管理，提高资金使用效益。分类推进事业单位改革。深化金融改革。积极引进国内外有实力的金融机构来唐展业。加快发展土地、技术、资本和劳动力等要素市场。加强市场监管体系建设，整顿规范市场秩序，建设法治市场、诚信市场和放心市场。在开放上，围绕产业优化升级，引导外资重点投向先进制造、高新技术、农产品加工、基础设施和现代服务业，着力提高对外开放层次。瞄准世界500强、国内大公司、大集团实施产业链招商、产业集群招商、品牌招商和园区招商，积极引进战略投资者。抓好高新区胶原蛋白等一批超千万美元重点利用外资项目的落实，提高合同履约率和外资到位率。办好第十届唐山中国陶瓷博览会，扩大品牌效应，赋予新的内涵，力争取得更大的成效。加快转变外贸增长方式，推动水泥、机械、电子元器件、陶瓷、纺织等优势产品出口向质量效益型转变。扩大关键设备、先进技术和短缺资源进口。支持有实力的企业“走出去”，承揽建设工程，开展矿产等重要资源合作开发。加强与国内大公司大集团和重点区域的战略合作，积极参与环渤海地区和京津冀都市圈产业分工协作，实现资源共享，优势互补，平等错位，共同发展。

(八)提高科技创新引领支撑能力。科学技术是提升综合竞争力的决定性因素。深入实施“十一五”科技发展规划，健全完善科技创新体系，推进创新型城市建设。加快科技创新步伐，积极推进传统产业高技术化、高新技术产业化、优势农业工业化和社会发展领域的科技进步。针对我市经济社会发展需求，重点实施装备制造集成创新与能力建设等21项重大科技创新工程，提高主导产业核心竞争力。加快打造科技创新基础平台、社会化科技服务平台、科技合作平台、科技人才和科技政策支撑平台，提升创新基础能力。加快建立以企业为主体、市场为导向、产学研相结合的技术创新体系，引导企业与高校、科研机构紧密合作，建立研发机构，增加科技投入，更好地发挥技术创新的主体作用。深入实施人才强市战略，加快培养和引进各类高层次人才、急需人才和适用人才，创新人才评价、激励、分配、保障机制，最大限度地激发各类人才的创业热情和创造活力。

(九)推动民营经济快速健康发展。坚持把民营经济作为最具活力和潜力的经济增长点，认真落实鼓励发展的各项政策措施，切实优化发展环境，进一步激活民营资本，推动民营经济扩张总量、提升质量。全市民营经济增加值增长20%以上。加强对中小企业的信贷支持，鼓励民营企业通过股份制改造、上市融资等措施，拓宽融资渠道。进一步加强信用担保体系、技术创新服务体系、创业辅导体系和人才培训体系建设，全市信用担保资金再增1.8亿元，达到8.6亿元。加强政策和信息引导，帮助民营企业正确把握投资导向，自觉执行国家产业政策和技术、环保、安全等市场准入标准。鼓励民营企业、中小企业在投资方向和项目选择上主动与全市产业结构调整对接，与生产力布局对接，与开发区、园区对接。积极推进民营企业制度创新、科技创新和管理创新，实现做大做强。完善创业激励机制，在全社会形成崇尚创业、竞相创业的浓厚氛围。

(十)坚持不懈地抓好安全生产。坚持安全第一、预防为主、综合治理的方针，深入开展安全生产“攻坚年、落实年”活动，全面加强消防、交通和生产安全工作，坚决防止重特大事故的发生。严格安全生产责任制，落实责任追究制。强化企业主体责任，加强安全管理制度建设，加大安全生产投入，抓好职工安全培训。健全安全生产监管体制，加强执法检查，综合运用经济、法律和行政手段，促进各项安全措施落实到位。推进安全生产长效机制建设，认真落实安全生产“三同时”和安全生产许可证制度，严格企业安全生产准入门槛。继续组织开展煤矿、非煤矿山、危化品、民爆器材、建筑施工等行业专项治理，深入持久地开展安全生产大检查，消除事故隐患。建立健全安全生产应急管理机制，提高防范和处置事故灾难的能力，维护人民群众的生命财产安全。

四、加强和谐唐山建设

发展的目的是为了人民群众的幸福安康。认真落实中央、省关于构建社会主义和谐社会的一系列重要举措，坚持把提高人民群众幸福指数作为一切工作的出发点和落脚点，深入实施“十大幸福工程”，建立幸福指数评价体系，切实解决好事关群众切身利益的问题，扎实推进和谐唐山建设。

(一)提升城市形象和现代化水平。城市是人民群众生活和创业的家园。围绕在冀东城市群建设中发挥好龙头作用，不断提升城市规划建设管理水平，努力把唐山建设成为适宜人居创业、充满人文关怀、有利于人的全面发展的现代化滨海城市。高起点、高站位搞好城市规划。坚持新思路、高站位、大手笔，按照“组团发展、西进东连、南扩北延、改造老区、建设新区、两带(南部沿海城市带、北部山前城市带)隆起”的总体布局，抓紧完成新一轮城市总体规划修编报批工作，抓好城市总体设计规划和城市景观、历史文化、交通、网络、绿化、水系等专项规划，进一步增强城市规划的科学性、指导性和文化内涵。加快规划曹妃甸滨海新城。高质量、高品位抓好城市建设。高标准推进机场新区开发建设，使其成为体现现代都市形象、具有现代城市产业特点的精品城区。进一步加大中心区老城改造力度，大力实施“退二进三”、“关旧上新”，优化城区产业布局。加快建设唐山大剧院、唐山“五个第一”博物馆、青少年宫、工人文化宫等一批标志性建筑和历史文化景观，提升城市品位。积极推进开滦国家矿山公园、地震遗址文化公园的规划建设，加强凤凰山、大城山、湾道山、陡河、青龙河、还乡河以及南湖、东湖“三山三河两湖”的环境综合改造。加快城市基础设施建设，完善公共交通体系，实施城市外环、唐丰快速路、城市出入口、垃圾处理、市区供热管网等一批重点市政工程，增强城市功能。加强房地产市场监管，重点发展商务用房、普通商品房和经济适用住房，促进房地产业健康有序发展。高效能、高水平加强城市管理。创新城市经营理念和管理机制，按照“先规划后建设、先征地后配套、先储备后开发、先地下后地上、先做环境后出让”的原则，加快推进城市资源资本化、资源配置市场化。加快供水、供热、供气等公用企(事)业单位改革步伐，推进市政公用设施建设和经营市场化运作。理顺城市管理体制，创新城市管理模式，实现管理重心下移，健全公共管理和服务功能。深入开展城市容貌综合整治，提高城市绿化美化亮化净化水平。

(二)加快城乡一体化进程。以统筹城乡发展为基本途径，以产业化提升农业、以工业化壮大实力、以城镇化改造农村，积极推动城乡规划一体化、建设一体化、产业一体化、制度一体化、公共服务一体化，促进大中小城市协调发展、城市和农村协调发展。坚持公共财政向农村倾斜、基础设施向农村延伸、公共服务向农村覆盖、现代文明向农村辐射，加大对新农村建设的支持力度。积极推进城镇化，优化城镇布局，因地制宜，发挥特色，加速产业向城镇集中、人口向城镇聚集。支持迁安、遵化等有条件的县(市)向中等城市迈进。加强城镇路、水、电、气、污水处理等基础设施建设和产业园区建设，为产业聚集和吸纳人口创造良好条件。加强城乡交通体系建设，抓好唐承高速二期、滨海公路、205国道改线等一批重点交通设施建设。积极实行城乡统一的户籍管理制度和就业制度。全市城镇化率达到49%以上。

(三)优先发展教育事业。以农村为重点，均衡发展基础教育，推进农村中小学布局调整和学校标准化建设，巩固提高“普九”成果，进一步完善“以县为主”的教育管理体制。实施教育扶助工程，建立完善教育资助制度。优化普通高中教育资源，抓好唐山一中、遵化高级中学等优质高中建设。全市初中毕业生升学率达到90%。积极发展高等教育，支持煤炭医学院新校区、理工大学新校区建设和博士点申报工作，抓好师范学院北校区、唐山学院北校区二期建设项目。适应经济社会发展需求，坚持以就业为导向，大力发展职业教育，努力打造唐山高级劳动技工学校等一批在全省乃至全国具有一定影响力的特色职业学校。完善成人教育体系，积极发展民办教育。切实加强教师队伍建设。

(四)加快发展文化、卫生、体育等社会公共事业。文化事业，认真落实《唐山市建设文化大市规划纲要》，实施文化繁荣工程，大力弘扬冀东地域文化，推进文化创新，打造文化品牌，营造良好文化环境。深化文化体制改革，推进文化单位转机建制。加大投入力度，健全完善公共文化服务体系，谋划建设广电大厦、新博物馆、科技馆等一批重点文化基础设施。大力发展文化产业，建立财政引导资金，支持文化旅游、媒体传播、演出娱乐、文化体育、民间艺术等文化主导产业发展。加强文化市场监管，抓好文化遗产、重点文物资源保护利用。推动社会、企业、乡村、家庭文化建设，丰富和活跃群众文化生活。卫生事业，着眼于缓解群众看病贵看病难问题，突出农村卫生、城市社区卫生、公共卫生服务三个重点，深化医疗卫生体制改革，实施全民健康工程，增强卫生保障能力和服务水平。抓好农村新型合作医疗规范管理，提高参合率，扩大覆盖面，全市农民参合率达到93%以上。大力发展城市社区卫生服务，新增社区卫生服务中心8个，人口覆盖率达到75%。加大传染病预防控制力度，提高突发公共卫生事件应急处置能力。加快市工人医院外科手术病房楼等一批公共卫生设施项目建设。强化卫生执法监督，规范药品流通秩序，确保人民群众就医用药安全。体育事业，以承办第十二届省运会为契机，广泛开展全民健身运动，提高竞技体育水平，积极发展体育产业。继续抓好人口和计划生育工作，稳定低生育水平，提高人口素质。与人民群众日益增长的物质文化生活水平相适应，加快广电、妇女、儿童、防震减灾、档案、气象等各项社会事业发展。

(五)千方百计扩大就业。就业是民生之本。实施扩大就业工程，全面落实促进就业的各项政策，支持自主创业、自谋职业和灵活就业，努力改善创业和就业环境。把扩大就业与改革发展、结构调整紧密结合起来，加快发展就业容量大的劳动密集型产业、非公有制经济、中小企业、服务业，加大公益性岗位开发力度，广辟就业渠道。完善就业公共服务体系建设，围绕主导产业和市场需求，加强就业、创业和再就业培训，年内免费培训下岗失业人员3万人以上，免费培训农民工9.8万人。加强就业指导和就业援助，重点做好下岗失业人员再就业、零就业家庭及就业困难人员就业、失地农民就业、高校毕业生就业、退伍军人就业与安置工作。加强劳动力市场监管，妥善处理劳动争议，维护劳动者合法权益。

(六)努力改善人民生活。在加快经济发展的基础上，努力增加城乡居民收入特别是中低收入者的收入，让老百姓的日子越过越好。建立农民增收减负长效机制，千方百计为农民增收广开门路。健全完善农民工工资保障机制，坚决杜绝拖欠农民工工资。落实企业最低工资制度，督导企业严格执行职工最低工资标准和工资指导线，确保职工工资收入正常增长。按照中央、省统一部署，做好公务员工资制度改革和规范津贴、补贴工作。推进事业单位收入分配改革。合理调整分配关系，加强税收征管，有效调节过高收入，坚决取缔非法收入，缓解城乡之间和部分社会成员之间收入差距扩大的趋势。实施安居工程，积极解决低收入家庭住房问题，加快建设中小套型普通商品房和经济适用住房，完善廉租房制度。全年经济适用住房开工面积87万平方米。继续组织实施为群众办实事工程。

(七)完善社会保障体系。进一步健全社会保障制度，实施社保扩面工程，以民营企业、农民工、个体工商户和灵活就业人员为重点，扩大社会保险覆盖面。强化社会保险基金征缴和监管，实现规范运作、按时足额发放。完善城镇职工基本医疗保险制度，做好路南、路北、开平、古冶四区医保纳入市级统筹管理工作，着手建立城镇居民医疗保险全覆盖制度。高度关注社会弱势群体，进一步完善社会救助体系。认真落实城乡居民最低生活保障制度，不断提高保障水平。完善医疗、教育、法律等专项救助办法，做到分类施保，应保尽保。广泛开展“真情暖万家扶贫济困”活动。大力发展社会福利、残疾人和老年事业，办好社会慈善事业。

(八)提高全社会文明水平。坚持把社会主义核心价值体系融入国民教育和精神文明建设全过程、贯穿现代化建设的各个方面，深入开展社会主义荣辱观教育，大力塑造并弘扬新时期唐山人文精神，在全社会形成共同理想，不断提高全社会的道德水准。以创建全国文明城市为目标，以文明行业、文明单位、文明社区、文明村镇、文明家庭创建为载体，组织开展多种形式的群众性精神文明创建和公民道德实践活动。加强诚信政府、诚信行业、诚信企业建设，不断提高全社会诚信水平。扎实开展“文明铸就和谐”活动，营造团结互助、和谐共处的社会风气，形成友爱和谐的人际关系，增强社会的亲和力和凝聚力。深入开展国防教育，加强军政军民团结，支持驻唐部队和民兵预备役建设。

(九)加强民主法制建设。自觉接受人民代表大会及其常委会的法律监督和工作监督，支持配合人民政协的民主监督、政治协商和参政议政，认真办理人大代表、政协委员的批评、意见、建议和提案，诚恳听取民主党派、工商联、无党派人士及各人民团体意见，确保行政权力公开透明运行。实施民主参与工程，加强基层政权建设和群众性自治组织建设，扩大基层民主，落实好各项公开制度，保证群众依法行使民主权利。拓宽了解民情、反映民意、集中民智的渠道，充分利用好每年12月份的“市民献计献策月”，广泛征询社会各界对政府工作的意见和诉求，不断提高决策科学化、民主化水平。加强和改进行政立法、行政执法及执法监督工作。深入开展“五五”普法宣传教育，提高全民特别是公职人员的法制观念和法律素质。

(十)维护社会稳定和谐。正确处理改革、发展、稳定的关系，实施平安创建工程。妥善处理新形势下的人民内部矛盾，深入细致地做好群众工作，认真解决好土地征用、城镇拆迁、水库移民、环境污染、企业改制、食品药品安全、涉法涉诉等方面群众反映强烈的问题。完善社会矛盾纠纷排查调处机制，统筹协调好各方面的利益关系。加强和改进信访工作，层层落实包案责任制和责任追究制，积极预防和妥善处置群体性事件，坚持依法办事，按政策办事，既帮助解决实际问题，又依法维护社会稳定。全面贯彻党的民族宗教政策，依法管理宗教事务，促进民族团结。切实加强公共安全管理，完善突发公共事件应急反应机制，提高安全防范水平。加强社会治安综合治理，依法严厉打击各种刑事犯罪，完善社会治安防控体系，保障人民群众安居乐业。

五、切实加强政府自身建设

新的形势和任务，对政府工作提出了新的要求。我们要牢记立党为公、执政为民的宗旨，大力弘扬胡锦涛总书记倡导的“八个方面”良好风气，实施政务创优工程，加快责任政府、法治政府、服务政府和效能政府建设，切实提高政府的执行力和公信力，努力开创我市改革发展新局面。

强化责任，全面履责。紧紧围绕抢抓新机遇、建设新唐山，进一步解放思想，创新思维，把思想和行动统一到市委重大决策部署上来。把处处争高、事事争好作为工作标准，始终保持奋发有为的精神状态，以无愧于时代的精神强化责任，以时不我待的精神抢抓机遇，以锲而不舍的精神真抓实干，以敢为人先的精神勇于创新，使各个方面的工作充满活力。抓好公务员队伍建设，加强学习，提高素质，不断增强各级各部门履职能力。加快推进政府职能转变，在更好地履行经济调节和市场监管的同时，切实加强社会管理和公共服务职能，更加注重维护社会公平，更加关注社会事业发展和解决民生问题。进一步理顺政府职能部门分工，全面落实工作责任制、行政问责制，做到权责统一，责任明确，严格考核，奖罚分明。

依法行政，规范作为。认真贯彻落实《国务院全面推进依法行政实施纲要》、《国务院办公厅关于全面推行行政执法责任制的若干意见》，严格按照法定权限和程序行使职权、履行职责，努力提高政府工作的规范化、法制化水平。深入实施《行政许可法》，积极推进行政审批制度改革。继续深化政务公开，建立健全社情民意反映制度和重大事项社会公示、听证和质询制度，自觉接受群众监督。将依法行政纳入行政机关和公务员考核指标体系，强化行政执法监督，严格落实行政执法责任制，坚决防止行政不作为、乱作为和违法施政现象发生。

转变作风，狠抓落实。大力弘扬求实精神和务实作风，严格按客观规律办事，做到科学决策、民主决策、依法决策。深入开展以“提速、提质，为人民、促发展”为主题的机关效能建设活动，降低行政成本，提高办事效率。大兴实干之风，少说多做，力戒浮躁，真正做到干事有激情、为民有真情、创业有热情，多出实绩、多留政绩，坚决不搞形式主义和劳民伤财的“形象工程”。下大力精简会议和文件，加强调查研究，落实目标责任制，把主要精力集中到抓重点、抓大事、抓主要矛盾上来，以责任明确的硬任务、真督实查的硬措施、赏罚分明的硬机制，扎扎实实把各项工作推向前进。

执政为民，廉洁从政。牢固树立正确的政绩观，强化民本意识，深怀爱民之心，恪守为民之责，善谋为民之策，多办利民之事，真正做到重大决策依民而定，工作措施应民而出，安危冷暖唯民而系。坚持深入基层、深入实际、深入群众，倾听群众呼声，体察群众疾苦，尽心竭力为群众办实事、办好事。牢记“两个务必”，发扬艰苦奋斗作风，坚决反对奢侈浪费。严格执行党风廉政建设责任制，加强理想信念教育、廉洁从政教育和警示教育，继续开展政务公开、清理专项资金、治理商业贿赂等工作，加大各种乱收费行为整治力度，坚决纠正各种损害群众利益的问题。加大违法违纪案件查办力度，严厉惩治腐败分子，以“为民、务实、清廉”的良好形象取信于民。

各位代表，今年的任务光荣而艰巨。让我们更加紧密地团结在以胡锦涛为总书记的党中央周围，在省委、省政府和市委的正确领导下，全面贯彻落实科学发展观，加压奋进，同心同德，和衷共济，扎实工作，为在科学发展道路上实现新跨越，为建设繁荣文明和谐新唐山而努力奋斗!
